# Supplementary material for: Amino acid, sugar, phenolic, and terpenoid profiles are capable of distinguishing Citrus tristeza virus infection status in citrus cultivars: Grapefruit, lemon, mandarin, and sweet orange
Source: PLoS One. 2022 May 10;17(5):e0268255. doi: 10.1371/journal.pone.0268255 (PMC9089872; doi:10.1371/journal.pone.0268255)
Supplement: S1 Appendix — Table A represents statistics for individual phenolics, Table B represents statistics for individual terpenoids, and Table C represents statistics for individual amino acids. (PDF) [file pone.0268255.s001.pdf]

Supplementary Table A. Summary of ANOVA statistics and effects for individual phenolic compounds. When compound is italicized then there was a significant effect for the test variable.

Test Variable	Compound	*F*	*p*	df1	df2	Differences by Tukey HSD*
Cultivar	*apigenin 6-C-glucosyl-7-O-(6-malyl-glucoside)*	101.221	0.000	5	162	Lisbon>all others; Oro Blanco>Murcott&Valencia;Navel>Valencia
	chrysoeriol-7-O-rutinoside	89.604	0.000	5	162	Oro Blanco>all others
	didymin	58.693	0.000	5	162	Lisbon>Oro Blanco>all others
	diosmin	85.177	0.000	5	162	Oro Blanco>Lisbon>all others
	eriocitrin	133.411	0.000	5	162	Lisbon>all others
	heptamethoxyflavone	91.466	0.000	5	162	Murcott>Lisbon&Minneola>Oro Blanco>Navel>Valencia
	hesperetin-7-O-glucoside	37.326	0.000	5	162	Valencia>all others except Navel; Murcott&Navel>Lisbon&Minneola&Oro Blanco
	hesperidin	23.547	0.000	5	162	Lisbon>Navel&Valencia>all others
	hexamethoxyflavone	43.755	0.000	5	162	Murcott>all others; Minneola>Minneola&Navel&Oro Blanco
	hexamethyl-O-quercetagetin	178.372	0.000	5	162	Murcott>Navel>Minneola&Valencia>Lisbon&Oro Blanco
	isorhoifolin-4-glucosde	52.629	0.000	5	162	Lisbon>Oro Blanco>all others
	isosinensetin	136.125	0.000	5	162	Murcott>Oro Blanco>Lisbon&Minneola>Navel&Valencia
	lucenin-2	207.556	0.000	5	162	Lisbon>Valencia>Minneola&Navel>Murcott&Oro Blanco
	lucenin-2 4-methyl ether	15.038	0.000	5	162	Lisbon>all others; Minneola&Murcott&Navel>Oro Blanco
	luteolin-7-rutinoside	16.018	0.000	5	162	Lisbon>all others; Oro Blanco>Murcott
	naringin	208.149	0.000	5	162	Lisbon>Oro Blanco>all others
	narirutin	67.207	0.000	5	162	Lisbon>Valencia>Navel>all others; Murcott>Oro Blanco
	natsudaidain	774.171	0.000	5	162	Murcott>all others; Minneola>Lisbon&Oro Blanco
	nobiletin	252.178	0.000	5	162	Murcott>Lisbon>all others; Minneola>Oro Blanco&Valencia;Navel>Oro Blanco
	rhoifolin	176.794	0.000	5	162	Oro Blanco>Lisbon>all others
	rutin	82.704	0.000	5	162	Oro Blanco>all others
	sinensetin	99.075	0.000	5	162	Murcott>Navel>Valencia>all others
	stellarin-2	89.334	0.000	5	162	Lisbon>all others

	tangeretin	206.332	0.000	5	162	Murcott>all others
	tetramethyl-O-scutellarein	217.536	0.000	5	162	Murcott>all others
	vicenin-2	42.217	0.000	5	162	Lisbon>Oro Blanco>all others; Murcott>Minneola
	*uk** flavanone 1*	36.484	0.000	5	162	Lisbon>all others>Oro Blanco
	uk flavone 1	97.771	0.000	5	162	Oro Blanco>Lisbon>all others; Navel&Valencia>Minneola
	uk flavone 2	10.948	0.000	5	162	Lisbon>Minneola&Murcott&Navel; Oro Blanco>Murcott
	uk flavonol methyl ester	35.221	0.000	5	162	Murcott>Minneola>all others
	uk polymethoxylated flavone	32.768	0.000	5	162	Murcott&Minneola>all others
	uk polymethoxylated flavone dimer	27.954	0.000	5	162	Oro Blanco>Lisbon>all others
Infection Status	apigenin 6-C-glucosyl-7-O-(6-malyl-glucoside)	0.182	0.834	2	162	
	chrysoeriol-7-O-rutinoside	0.954	0.387	2	162	
	didymin	1.077	0.343	2	162	
	diosmin	13.123	0.000	2	162	Healthy&Severe>Mild
	eriocitrin	0.805	0.449	2	162	
	heptamethoxyflavone	5.987	0.003	2	162	Mild>Severe
	hesperetin-7-O-glucoside	5.381	0.005	2	162	Healthy>Severe
	hesperidin	0.852	0.428	2	162	
	hexamethoxyflavone	0.118	0.889	2	162	
	hexamethyl-O-quercetagetin	0.471	0.625	2	162	
	isorhoifolin-4-glucoside	4.454	0.013	2	162	Mild>Severe
	isosinensetin	1.678	0.190	2	162	
	lucenin-2	0.996	0.372	2	162	
	lucenin-2 4-methyl ether	5.606	0.004	2	162	Mild>Severe
	luteolin-7-rutinoside	8.945	0.000	2	162	Mild>Severe

	naringin	2.375	0.096	2	162	
	narirutin	2.562	0.080	2	162	
	natsudaidain	2.136	0.121	2	162	
	nobiletin	20.984	0.000	2	162	Mild>Severe
	rhoifolin	3.088	0.048	2	162	Healthy>Mild
	rutin	0.974	0.380	2	162	
	sinensetin	0.022	0.978	2	162	
	stellarin-2	3.419	0.035	2	162	Severe>Mild
	tangeretin	0.680	0.508	2	162	
	tetramethyl-O-scutellarein	0.164	0.849	2	162	
	vicenin-2	2.156	0.119	2	162	
	uk flavanone 1	4.430	0.013	2	162	Severe>Mild
	uk flavone 1	3.912	0.022	2	162	Severe>Healthy
	uk flavone 2	17.035	0.000	2	162	Health&Mild>Severe
	uk flavonol methyl ester	10.484	0.000	2	162	Healthy>Mild&Severe
	uk polymethoxylated flavone	5.539	0.005	2	162	Severe>Mild
	uk polymethoxylated flavone dimer	1.392	0.251	2	162	
Interaction	apigenin 6-C-glucosyl-7-O-(6-malyl-glucoside)	1.766	0.071	10	162	
	chrysoeriol-7-O-rutinoside	0.589	0.821	10	162	
	didymin	0.457	0.915	10	162	
	diosmin	6.331	0.000	10	162	
	eriocitrin	0.789	0.639	10	162	
	heptamethoxyflavone	2.984	0.002	10	162	
	hesperetin-7-O-glucoside	1.515	0.138	10	162	
	hesperidin	0.776	0.652	10	162	
	hexamethoxyflavone	0.558	0.846	10	162	

hexamethyl-O-quercetagetin	1.268	0.252	10	162
isorhoifolin-4-glucosde	0.898	0.536	10	162
isosinensetin	4.718	0.000	10	162
lucenin-2	1.204	0.292	10	162
lucenin-2 4-methyl ether	1.685	0.088	10	162
luteolin-7-rutinoside	3.892	0.000	10	162
naringin	0.801	0.627	10	162
narirutin	1.209	0.289	10	162
natsudaidain	2.285	0.016	10	162
nobiletin	4.722	0.000	10	162
rhoifolin	3.046	0.001	10	162
rutin	1.047	0.407	10	162
sinensetin	1.450	0.163	10	162
stellarin-2	1.459	0.159	10	162
tangeretin	2.627	0.005	10	162
tetramethyl-O-scutellarein	0.347	0.967	10	162
vicenin-2	1.039	0.413	10	162
uk flavanone 1	0.813	0.616	10	162
uk flavone 1	1.929	0.045	10	162
uk flavone 2	3.987	0.000	10	162
uk flavonol methyl ester	2.311	0.014	10	162
uk polymethoxylated flavone	3.022	0.002	10	162
uk polymethoxylated flavone dimer	3.578	0.000	10	162

* If ANOVA was significant ($p < 0.05$)
** uk = unknown/unidentified

Supplementary Table B. Summary of ANOVA statistics and effects for individual terpenoid compounds. When compound is italicized then there was a significant effect for the test variable.

Test Variable	Compound	*F*	*p*	df1	df2	Differences by Tukey HSD*
Cultivar	*α-phellandrene*	1.443	0.212	5	162	Valencia>Minneola&Murcott&Navel; Oro Blanco>Navel
	α-pinene	258.822	0.000	5	162	Murcott>Minneola>all others; Lisbon&Navel>Valencia
	α-terpinene	52.562	0.000	5	162	Murcott>all others>Minneola
	α-terpinolene	94.931	0.000	5	162	Murcott>Navel>all others>Oro Blanco
	β-caryophellene	85.807	0.000	5	162	Lisbon>Murcott>all others
	β-myrcene	48.691	0.000	5	162	Navel&Lisbon>Oro Blanco >Murcott&Valencia>Minneola
	cis-β-ocimene	172.051	0.000	5	162	Murcott>Minneola&Navel>all others
	trans-β-ocimene	72.118	0.000	5	162	Murcott&Lisbon>all others; Navel>Valencia
	β-pinene	89.495	0.000	5	162	Murcott>Lisbon>Minneola>all others
	δ-3-carene	98.465	0.000	5	162	Navel>Valencia>Lisbon>all others
	γ-terpinene	985.287	0.000	5	162	Murcott>Minneola>all others
	bornyl acetate	137.765	0.000	5	162	Lisbon>Navel>all others
	camphene	13.106	0.000	5	162	Murcott&Lisbon>all others
	carvone	75.087	0.000	5	162	Lisbon>Navel>all others
	citral	86.814	0.000	5	162	Lisbon>all others; Navel>all others except Valencia
	damasceone isomer 1	64.586	0.000	5	162	Lisbon>Murcott>all others
	damasceone isomer 2	250.944	0.000	5	162	Murcott>Navel>all others>Lisbon
	limonene	97.727	0.000	5	162	Lisbon>all others
	linalool	300.808	0.000	5	162	Murcott>Minneola>Navel>Valencia>Lisbon&Oro Blanco
	myrtenol	64.168	0.000	5	162	Lisbon>Navel>Murcott>all others
	para-cymene	78.966	0.000	5	162	Murcott>Minneola>all others
	pulegone	137.677	0.000	5	162	Minneola>all others
	uk terpenoid 1	544.827	0.000	5	162	Murcott>Minneola>all others; Navel>Lisbon&Valencia
	uk terpenoid 2	82.111	0.000	5	162	Navel&Oro Blanco>Valencia>all others
	uk terpenoid 3	15.142	0.000	5	162	Valencia>all except Navel; Lisbon&Navel&Oro Blanco >Minneola&Murcott
Infection Status	α-phellandrene	2.039	0.134	2	162	
	α-pinene	0.852	0.428	2	162	
	α-terpinene	8.703	0.000	2	162	Healthy&Mild>Severe
	α-terpinolene	0.819	0.443	2	162	

	β-caryophellene	0.979	0.378	2	162	
	β-myrcene	1.120	0.329	2	162	
	cis-β-ocimene	0.510	0.601	2	162	
	trans-β-ocimene	1.142	0.322	2	162	
	β-pinene	5.850	0.004	2	162	Mild>Healthy&Severe
	δ-3-carene	2.202	0.114	2	162	
	γ-terpinene	2.193	0.115	2	162	
	bornyl acetate	0.180	0.835	2	162	
	camphene	1.980	0.141	2	162	
	carvone	2.116	0.124	2	162	
	citral	1.876	0.156	2	162	
	damasceone isomer 1	1.505	0.225	2	162	
	damasceone isomer 2	0.532	0.589	2	162	
	limonene	0.343	0.710	2	162	
	linalool	3.445	0.034	2	162	Mild>Healthy
	myrtenol	1.392	0.251	2	162	
	para-cymene	3.699	0.027	2	162	Severe>Mild
	pulegone	2.659	0.073	2	162	
	uk terpenoid 1	1.167	0.314	2	162	
	uk terpenoid 2	2.340	0.100	2	162	
	uk terpenoid 3	0.000	1.000	2	162	
Interaction	α-phellandrene	1.032	0.419	10	162	
	α-pinene	1.093	0.371	10	162	
	α-terpinene	8.313	0.000	10	162	
	α-terpinolene	5.813	0.000	10	162	
	β-caryophellene	0.925	0.512	10	162	
	β-myrcene	0.916	0.520	10	162	
	cis-β-ocimene	1.107	0.360	10	162	
	trans-β-ocimene	1.056	0.399	10	162	
	β-pinene	1.694	0.086	10	162	
	δ-3-carene	0.692	0.731	10	162	
	γ-terpinene	1.714	0.081	10	162	
	bornyl acetate	0.630	0.787	10	162	
	camphene	1.113	0.356	10	162	

carvone	1.733	0.077	10	162
citral	1.586	0.115	10	162
damasceone isomer 1	1.449	0.163	10	162
damasceone isomer 2	0.161	0.998	10	162
limonene	0.425	0.933	10	162
linalool	4.033	0.000	10	162
myrtenol	1.037	0.415	10	162
para-cymene	2.809	0.003	10	162
pulegone	2.722	0.004	10	162
uk terpenoid 1	1.021	0.428	10	162
uk terpenoid 2	0.936	0.502	10	162
uk terpenoid 3	1.104	0.362	10	162

* If ANOVA was significant ($p < 0.05$)

Supplementary Table C. Summary of ANOVA statistics and effects for individual amino acid compounds. When compound is italicized then there was a significant effect for the test variable.

Test Variable	Compound	*F*	*p*	df1	df2	Differences by Tukey HSD*
Cultivar	*alanine*	5.233	0.000	5	161	Murcott>all others except Navel
	asparagine	4.985	0.000	5	161	Minneola>all others except Navel; Navel>Lisbon&Valencia
	aspartic acid	19.045	0.000	5	161	Minneola&Murcott>all others; Lisbon&Navel>Oro Blanco
	glutamic acid	12.655	0.000	5	161	All>Oro Blanco; Navel>Minneola&Valencia
	glutamine	171.071	0.000	5	161	Murcot>Lisbon&Minneola>all others
	glycine	7.690	0.000	5	161	Valencia>all others; Lisbon&Murcott>Oro Blanco
	histidine	100.030	0.000	5	161	Murcott>Minneola&Navel&Valencia>Lisbon&Oro Blanco
	isoleucine	22.143	0.000	5	161	Murcott>all others; All>Oro Blanco
	leucine	26.483	0.000	5	161	Murcott>all others; All>Oro Blanco
	lysine	76.254	0.000	5	161	Murcott>Minneola>Lisbon&Navel&Valencia>Oro Blanco
	methionine	63.852	0.000	5	161	Murcott>Valencia&Minneola>Navel>Lisbon&Oro Blanco
	ornithine	139.636	0.000	5	161	Minneola>Murcott>all others
	phenylalanine	39.955	0.000	5	161	Murcott>all others; Minneola&Valencia>All Others Except Murcott; Minneola>Lisbon
	proline	12.522	0.000	5	161	Murcott>all others except Navel; All>Oro Blanco
	serine	77.288	0.000	5	161	Murcott>Navel>Minneola&Valencia>Lisbon>Oro Blanco
	threonine	66.831	0.000	5	161	Murcott>all others; All except Lisbon>Oro Blanco; Minneola>Lisbon
	tryptophan	17.985	0.000	5	161	Murcott&Minneola>all others
	tyrosine	185.333	0.000	5	161	Murcott>Minneola>Navel&Valencia>Lisbon>Oro Blanco
	valine	15.500	0.000	5	161	Murcott>all others; All except Navel>Oro Blanco
Infection Status	alanine	2.267	0.107	2	161	
	asparagine	1.141	0.322	2	161	
	aspartic acid	1.362	0.259	2	161	
	glutamic acid	19.423	0.000	2	161	Severe>Mild&Healthy
	glutamine	3.824	0.024	2	161	None
	glycine	8.223	0.000	2	161	Severe>Mild
	histidine	0.482	0.619	2	161	
	isoleucine	1.397	0.250	2	161	

	leucine	1.542	0.217	2	161	
	lysine	1.040	0.356	2	161	
	methionine	3.105	0.048	2	161	None
	ornithine	1.410	0.247	2	161	
	phenylalanine	6.089	0.003	2	161	Severe>Mild
	proline	20.693	0.000	2	161	Severe>Mild&Healthy
	serine	2.560	0.080	2	161	
	threonine	0.917	0.402	2	161	
	tryptophan	11.725	0.000	2	161	Severe>Mild
	tyrosine	3.936	0.021	2	161	Severe>Mild
	valine	1.907	0.152	2	161	
Interaction	alanine	1.233	0.274	10	161	
	asparagine	0.593	0.818	10	161	
	aspartic acid	1.587	0.115	10	161	
	glutamic acid	1.661	0.094	10	161	
	glutamine	2.651	0.005	10	161	
	glycine	1.164	0.319	10	161	
	histidine	0.667	0.754	10	161	
	isoleucine	2.665	0.005	10	161	
	leucine	2.255	0.017	10	161	
	lysine	3.423	0.000	10	161	
	methionine	1.861	0.054	10	161	
	ornithine	1.315	0.226	10	161	
	phenylalanine	2.012	0.035	10	161	
	proline	2.160	0.023	10	161	
	serine	1.625	0.104	10	161	
	threonine	1.926	0.045	10	161	
	tryptophan	1.710	0.082	10	161	
	tyrosine	3.202	0.001	10	161	
	valine	1.985	0.038	10	161	

* If ANOVA was significant
(p < 0.05)
