# Supplementary material for: Amino acid, sugar, phenolic, and terpenoid profiles are capable of distinguishing Citrus tristeza virus infection status in citrus cultivars: Grapefruit, lemon, mandarin, and sweet orange
Source: PLoS One. 2022 May 10;17(5):e0268255. doi: 10.1371/journal.pone.0268255 (PMC9089872; doi:10.1371/journal.pone.0268255)
Supplement: Attachment — Submitted filename: Response to Reviewers.docx [file pone.0268255.s004.docx]

# **Amino acid, sugar, phenolic, and terpenoid profiles are capable of distinguishing *Citrus tristeza virus* infection status in citrus cultivars: grapefruit, lemon, mandarin, and sweet orange**

## **Editor Comment:**

In their response, the authors claimed they changed the main title into "Amino acid, sugar, phenolic, and terpenoid profiles are capable of distinguishing Citrus tristeza virus infection status in citrus cultivars grapefruit, lemon, mandarin, and sweet orange” as suggested by Reviewer #1. However, they missed to do so.

Please add a colon after "cultivars".

Author Response: We apologize for the oversight. The title has been changed as suggested, including adding the colon after “cultivars”.
