# Supplementary material for: Amino acid, sugar, phenolic, and terpenoid profiles are capable of distinguishing Citrus tristeza virus infection status in citrus cultivars: Grapefruit, lemon, mandarin, and sweet orange
Source: PLoS One. 2022 May 10;17(5):e0268255. doi: 10.1371/journal.pone.0268255 (PMC9089872; doi:10.1371/journal.pone.0268255)
Supplement: Attachment — Submitted filename: Reviewer comments KKB -1-3-22.docx [file pone.0268255.s002.docx]

This manuscript Aminoacid,sugar,phenolic,andterpenoidprofilesarecapableofdistinguishingCitrustristezavirusinfectionstatusingrapefruit,lemon, mandarin,and sweet orange leaves” is well organized and provides an assessment of changes the level of amino acid, sugar, phenolic, and terpenoid in healthy and CTV-infected grapefruit, lemon, mandarin, and sweet orange cultivars. The results could improve understanding about citrus-CTV interactions and biochemistry in CTV infected plant.The study has shown novelty and usefulness of the data for distinguishing infection by evere and mild CTV in infected citrus trees, thus this MS could be published.However, I think that editing the paper is needed.

The title should be changed like “Aminoacid,sugar,phenolic,andterpenoidprofilesarecapableofdistinguishingCitrus tristezavirusinfectionstatusin citrus cultivars grapefruit, lemon, mandarin,andsweetorange” tleile

Line 24-30: In abstract portion ( However, canonical discriminant analysis (CDA) utilizing profiles of individual amino acids, terpenoids, or phenolics was able to correctly match leaf samples to specific citrus varieties and identify their infection status with good accuracy. This analysis reliably distinguished citrus infected with mild CTV strains from those infected with severe CTV strains. Collectively, this study reveals biochemical patterns associated with severity of CTV infections that can potentially be utilized to help identify in-field CTV infections of economic relevance.)- Should be more clear

Line 55-76: Introduction portion Para 3 and 4 should be merged and to be shorten. Symptomatology should not be so highlighted as correlation with the symptom severity and metabolic changes was not shown clearly in this paper

References cited in the text is not accordingly the journal pattern-should be corrected (numbering?)

Bibliography list of references should be accordingly the PLOSONE pattern. Consult any recent reprint of PLOSONE and correct it accordingly.

Line 441: “Ultimately, this study provides unique insight into the biochemical mechanisms associated with CTV, establishing distinct metabolic patterns associated with the differential 443 severity of CTV strain?.--biochemical mechanisms- I feel this terms are not required here.

Line 443: “This study shows CDAs are a useful tool for discernment of trends and 444 patterns in biochemical shifts that are otherwise not readily observable.” CDAs could be useful… as more standardization is needed. It has tremendous potentiality to distinguishing the infection caused by severe and mild strain as the antisera, developed as specific (called) to severe and mild CTV, could not always work. As in introduction it was mentioned “However, 59 a recent study observing MCA13+ and MCA13 negative (MCA13-) strains infecting trees in the 60 same orchards did not observe differences in symptoms or yields (Yokomi et al. 2020).
